# Urinary CD4^+^ T helper cells are a potential biomarker for tubulointerstitial nephritis in Sjögren’s disease

**DOI:** 10.1038/s41598-025-34685-x

**Published:** 2026-02-11

**Authors:** Viona Laas, Frederic Christian Feindt, Pascal Böse, Tom Zimmermann, Pavels Klimicevs, Julia Hagenstein, Laura-Isabell Ehnold, Georg R. Herrnstadt, Matthias T. Warkotsch, Hans-Joachim Paust, Ulf Panzer, Christian F. Krebs, Tobias B. Huber, Thorsten Wiech, Oliver M. Steinmetz, Simon Melderis

**Affiliations:** 1 III. Department of Medicine, University Medical Center Hamburg-Eppendorf, Martinistrasse 52, 20246 Hamburg, Germany; 2 Department of Pathology, Section of Nephropathology, University Medical Center Hamburg-Eppendorf, Hamburg, Germany; 3 Hamburg Center for Kidney Health (HCKH), University Medical Center Hamburg-Eppendorf, Hamburg, Germany

**Keywords:** Biomarkers, Diseases, Immunology, Medical research, Nephrology

## Abstract

Sjögren’s Disease (SjD) is the most common connective tissue disease. An estimated 5–27% of patients have kidney involvement, usually in the form of tubulointerstitial nephritis (TIN). To date, there are no validated biomarkers for either diagnosis or assessment of therapeutic response. Since one of the hallmarks of TIN is leukocyturia, we aimed at investigating the potential role of urinary leukocytes as a diagnostic tool for SjD-TIN. We prospectively recruited 13 patients with SjD, that underwent kidney biopsy for suspected TIN. A multicolor flow cytometry panel was established to quantify 8 different urinary leukocyte populations. Our analyses showed, that urinary CD4^+^ T_H_ cells are increased in patients with SjD-TIN and can differentiate precisely between SjD-TIN and SjD-patients with other non-inflammatory kidney pathologies (SjD-CKD). In contrast, no other urinary cell population or clinical marker was able to differentiate between SjD-TIN and SjD-CKD. Furthermore, we found a very strong correlation between urinary CD4^+^ T_H_ cells and histological severity of TIN. Finally, during longitudinal follow up of 8 SjD-TIN patients undergoing immunosuppressive therapy, we saw a large and significant drop in urinary CD4^+^ T_H_ cells in parallel with clinical and histological improvement. Taken together our data suggest, that urinary CD4^+^ T_H_ cells might be a novel biomarker for diagnosis and follow up of SjD-TIN and might help to guide treatment decisions.

**Supplementary Information:**

The online version contains supplementary material available at 10.1038/s41598-025-34685-x.

## Introduction

Sjögren’s disease (SjD, formerly Sjögrens’ Syndrome) is the most common connective tissue disease worldwide with a prevalence of approximately 61 per 100.000 people^1^. A large proportion of patients only has sicca symptoms (e.g. dry eyes & dry mouth) but a significant proportion (30–40%) develop extra-glandular disease manifestations^2^. The prevalence of kidney involvement varies from low estimates of 5% up to 27% when comprehensive screening for kidney pathology is performed^3–5^. Tubulointerstitial nephritis (TIN) is the most common form of kidney involvement in SjD^3,6–12^, often leading to chronic kidney disease with high morbidity in the affected patients. The pathophysiology of SjD-TIN is largely unknown and thus the therapy is uncertain and only based on expert recommendations and retrospectively analysed cohorts. The histological picture of SjD-TIN is characterized by a renal inflammatory infiltrate, predominantly consisting of lymphocytes, including a high percentage of plasma cells.

So far, no validated biomarkers exist to help with the diagnosis of SjD-TIN. In the past, a number of markers have been evaluated for diagnosis of TIN in general, as in particular urinary excretion of the chemokine CXCL9 as well as cytokines IL-9 and TNF-α^13,14^. However, to our knowledge all previous studies have either looked specifically at drug-induced forms of TIN, or at an unspecified basket of different TIN etiologies. Given the stark immunological differences between SjD and drug hypersensitivity reactions or other underlying TIN pathologies such as sarcoidosis and inflammatory bowel disease, the validity of the above mentioned results for SjD-TIN are uncertain^13–15^.

A particular hallmark of TIN is invasion of lymphocytes across the renal tubular basement-membrane into the renal tubular epithelia by unknown mechanisms, called tubulitis. From this anatomical niche, leukocytes can transmigrate with relative ease into the tubular lumen and gain access to the urine. Leukocyturia has thus long been recognized as one of the hallmarks of TIN^16,17^. It has been shown that detailed quantification of urinary leukocytes can give some insight into renal immune-pathophysiology. Enghard et al. have pioneered analysis of leukocyturia via flow cytometry (FC) in patients with glomerulonephritis (GN) and kidney transplant rejection^18–22^. For example, they could show, that patients with active lupus nephritis have increased absolute urinary CD4^+^ T_H_ cells and that increased urinary CD4^+^ T_H_ cells predict renal flares in anti-neutrophil cytoplasmic antibodies (ANCA)-GN patients. Along the same line, they could show that CD8^+^HLA-DR^+^ T cells were enriched in the urine of kidney transplant recipients who suffered acute T cell mediated rejection. This body of work shows, that leukocyturia can be a valuable tool in assessing the activity of inflammatory kidney diseases.

However, it is not known, whether leukocyturia also reflects the presence of TIN or SjD-TIN in particular. Furthermore, it has thus far not been studied, if the degree of leukocyturia reflects the degree of histological inflammation of the kidney. Lastly and most importantly, it remains unclear whether leukocyturia might also be used longitudinally as a biomarker indicating therapeutic response.

We thus aimed to close these gaps and evaluate the clinical use of detailed flow cytometric analysis of leukocyturia in patients with SjD-TIN.

## Materials and methods

### Patient cohort

We performed a single centre study at the III. Department of Medicine, University Medical Center Hamburg-Eppendorf, Hamburg, Germany (UKE). From January 2021 to December 2024, we prospectively screened all patients with Sjögren’s Disease who underwent kidney biopsy for suspected kidney disease. Patients, that had an escalation of therapy shortly before the kidney biopsy were excluded from the study. After informed consent was obtained, patients were included in the study if they had a kidney biopsy and a urine sample prior to biopsy. In four of the SjD-TIN patients a repeat kidney biopsy during the maintenance phase of treatment was performed for clinical indication at a median of 549 days after treatment initiation.

Our primary cohort for this study were SjD patients with biopsy proven TIN (SjD-TIN). All patients fulfilled the ACR/EULAR classification criteria for SjD^23^. Patients were followed up and treated in our specialized outpatient clinic at the discretion of the treating physicians (S.M. and O.M.S.). All treatment decisions were made prior to and independent of data generated for research purposes. Routine clinical data, as well as urine for longitudinal flow cytometric analyses were collected during routine clinical follow up.

Control groups were defined as follows:

(i) SjD-patients with chronic kidney disease (SjD-CKD). These patients with diagnosed and classified SjD were biopsied for suspected kidney disease with the biopsy showing changes consistent with CKD but no TIN or other SjD related kidney pathologies.
(ii) Patients with biopsy proven chronic kidney disease (CKD). These were patients referred to our outpatient clinic between January 2021 and December 2024, that were biopsied for suspected TIN of unknown etiology, but the biopsy only showed changes consistent with CKD. None of these patients had SjD.

Patients from groups (i) and (ii) were only included in the study, if they had a kidney biopsy and a urine sample prior to biopsy.

(iii) Patients with urinary tract infections (UTI). This group was defined as patients with clinical symptoms consistent with UTI and a positive urine culture. Patients were recruited at the UKE between January 2021 and December 2024.
(iv) Healthy controls (HC) without any evidence of kidney disease or SjD. Absence of kidney disease was determined by urine dipstick test and history.

For all SjD patients the EULAR Sjögren’s syndrome disease activity index (ESSDAI) was determined immediately prior to kidney biopsy^24^. All patients gave informed consent. The study was approved by the ethics committee of the Hamburg Medical Chamber (registration numbers PV4806, PV5026 and PV5822) and was conducted according to the ethics guidelines at our institution and the Declaration of Helsinki.

### Routine clinical data

All clinical data was collected as part of routine clinical care. Patients with TIN were initially treated as inpatients and followed up in our specialized outpatient clinic. Routine samples (blood and urine) were analysed in the department for diagnostics of the UKE. Urinary sediments were performed as part of the clinical routine.

### Histological analysis of kidney biopsies

Routine histopathological analysis was blinded as to the results from flow cytometry and clinical information and was performed by T.W., an experienced renal histopathologist. No validated classification system for the severity of SjD-TIN exists. For cellular tubulointerstitial kidney transplant rejection the BANFF classification has long been used^25^. Since transplant rejection and TIN are histologically very similar, we applied a modified BANFF (mBANFF) classification to our biopsies. Our mBANFF classification is based on six BANFF criteria and two additional scores (see below). The BANFF criteria are: percentage of non-scarred renal cortex area affected by inflammation (i), tubulitis (t), percentage of total renal cortex area, including areas of interstitial fibrosis and tubular atrophy (IFTA) affected by interstitial inflammation (ti), percentage of scarred renal cortex area affected by inflammation (i-IFTA), percentage of interstitial area affected by fibrosis (ci) and percentage of interstitial area with tubular atrophy (ct).

Given that the interstitial infiltrate in SjD-TIN is often rich in plasma cells, we additionally assessed the percentage of plasma cells among all inflammatory cells (pc). For all categories a score from 0 to 3 points is applied.

In the case of TIN, tubulitis is the most relevant parameter to assess acute inflammatory activity. In the BANFF System tubulitis (t) is scored by determining the number of mononuclear cells per 10 tubular epithelial cells, which is the average number of epithelial cells per tubular cross section. Tubulitis must be present in at least 2 foci and the most severely affected tubule determines the score. The t-score is dependent on the number of cells: t0—No mononuclear cells in tubules or presence of a single focus of tubulitis only. t1—Foci with 1 to 4 mononuclear cells/tubular cross section (or 10 tubular cells). t2—Foci with 5 to 10 mononuclear cells/tubular cross section (or 10 tubular cells). t3—Foci with > 10 mononuclear cells/tubular cross section or the presence of ≥ 2 areas of tubular basement membrane destruction accompanied by i2/i3 inflammation and t2 elsewhere^26^. For a more precise analysis of tubulitis, we additionally recorded the exact number of mononuclear cells per tubular cross section (i.e. 10 tubular cells on average), underlying the classification into the above mentioned four t-scores (t-exact).

### Urinary flow cytometry

45 ml of urine were collected immediately after voiding and processed within 1–3 h of voiding and diluted with 5 ml of PBS + 0.5% BSA + 1% penicillin/streptomycin (pen/strep). Samples were placed on ice (4 °C) for transportation and were centrifuged at 350 g for 10 min at 4 °C. Cell pellets were resuspended in 2 ml PBS + 0.5% BSA + 1% pen/strep and filtered through a 40-µm nylon mesh. After spinning the samples for 5 min at 350 g at 4 °C unspecific binding sites were blocked with 10% human IgG in PBS + 0.5% BSA + 1% pen/strep for 20 min and cells were transferred to a 96-well-plate. The plate was centrifuged at 360 g for 2 min at 4 °C and the supernatant was discarded. Cells were surface stained for 20 min at 4 °C. The surface panel included CD45-BV510, CD3-AF700, CD4-BV785, CD8a-PerCP, CD19-Pb, CD56-BV605, CD11b-FITC, CD66b-PE, CD16-APC, CD14-BV711 and Siglec8-PeCy7. Cells were washed twice with 150 µl PBS and centrifuged at 360 g for 2 min at 4 °C. To identify dead cells, Live/Dead staining was performed with 0,1% NIR for 8 min at 4 °C. After washing twice, cells were fixed in 3,65% paraformaldehyde (PFA) for 15 min at 4 °C, washed twice and resuspended in 150 µl PBS. The complete 150 µl cell suspension was analysed by flow cytometry, ensuring, that all cells, originally in the 45 ml urine sample were accounted for. Experiments were performed on a BD FACS Symphony A3 (BD Biosciences, Heidelberg, Germany). Data was analysed using Flow Jo Software (v.10.10, FlowJo LLC, Ashland, Oregon, USA).

The following buffer/reagents were used: Phosphate Buffered Saline without Calcium and Magnesium (catalog number: 14190–094, 500 ml), penicillin/streptomycin solution (15070063, 100 ml), Trypan Blue (15250061, 100 ml) and near-IR fluorescent reactive dye (L10119A) were purchased from Thermo Fisher Scientific (Waltham, Massachusetts, USA). AutoMACS Running Buffer (130-091-221, 1500 ml) and human FcR Blocking Reagent (130-059-901) were purchased from Miltenyi Biotec (Bergisch Gladbach, Germany). Bovine serum albumin (A7030-50 g), formaldehyde solution (F8775-25 ml) and Trizol (T9424-100 ml) were purchased from Sigma-Aldrich (St. Louis, Missouri, USA).

For the surface staining the following antibodies were used: CD45-BV510 (304036, HI30), CD3-AF700 (300424, UCHT1), CD4-BV785 (300553, RPA-T4), CD8a-PerCP (301030, RPA-T8), CD19-Pb (302224, HIB19), CD56-BV605 (318333, HCD56), CD66b-PE (392903, 6/40c), CD16-APC (302011, 3G8), CD14-BV711 (301837, M5E2) and Siglec-8-PeCy7 (347111, 7C9) were purchased from BioLegend (San Diego, CA, USA). CD11b-FITC (557396, M1/70) was purchased from BD Biosciences (Franklin Lakes, New Jersey, USA).

### Urinary creatinine

For determination of urinary creatinine of the healthy controls a commercial creatinine detection kit was used (Hengler Analytik, Steinbach, Germany, catalog number: 114444). 10 μl of standard (Sigma, St. Louis, Missouri, USA, C4255-10 g) and urine samples were pipetted into a 96-well-plate. Creatinine concentrations were measured according to the kit instructions using a microplate reader (EL808, BioTek Instruments, Vermont, USA). For assessment of absolute urinary cells, we normalized the concentration of cells in the urine to the concentration of creatinine in the urine with final units cells/mg of urinary creatinine.

### Statistical methods and study design

Based on preliminary data and a primary outcome of differences in relative urinary CD4^+^ T_H_ cells of SjD-TIN and SjD-CKD patients, an a priori power analysis for mean differences was performed with G*Power (version 3.1.9.7) and parameters of: effect size (d) = 2.1, α err prob. = 0.05, power (1-β err prob.): 0.8, allocation ratio 1/1. We calculated a power of 83% to detect a significant difference between urinary CD4^+^ T_H_ cells of SjD-TIN and SjD-CKD patients with a sample size of 5 patients per group. Results are presented as mean ± standard error of the mean (SEM) if not specifically specified otherwise. To compare two groups, the Student’s t-test and the Mann–Whitney test were used where appropriate. One-way ANOVA and Kruskal–Wallis test (both with Dunnett’s multiple comparison test) were used to compare multiple groups. Multiple comparison was done by comparing SjD-TIN with all other groups. For correlation of two parameters Pearson’s or Spearman’s correlation coefficient was used where appropriate. Statistical significance was determined by P-value: < 0.05 = *, < 0.01 = ** and < 0.001 = ***.

## Results

### Histopathological features of kidney involvement in SjD

**Fig. 1:**
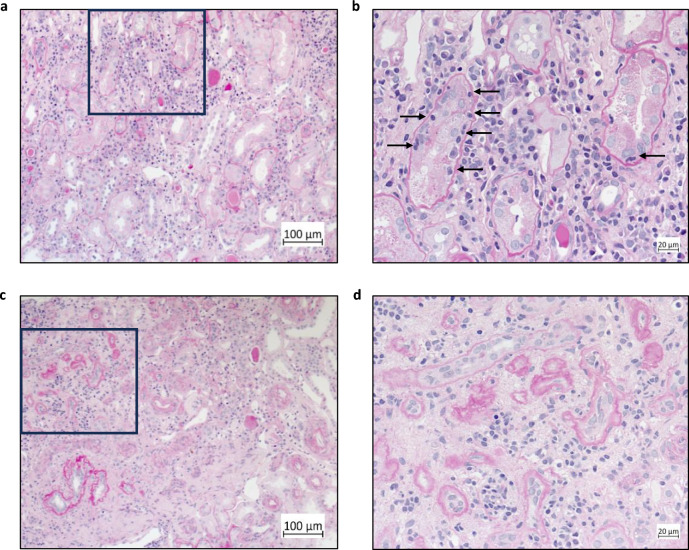
Histopathology of SjD-TIN and SjD-CKD. (**a**, **b**) Representative PAS-stained kidney sections with magnification of 100x (**a**) and 400x ((**b**), magnified from black square in (**a**)) from a SjD-TIN patient. A dense interstitial infiltrate and significant tubulitis (black arrows), both hallmarks of TIN, can be seen. (**c**, **d**) Representative PAS-stained kidney sections with magnification of 100x (**c**) and 400x ((**d**), magnified from black square in (**c**)) from a SjD-CKD patient, showing interstitial fibrosis and tubular atrophy. Interstitial infiltration of leukocytes is only found in fibrotic areas, while there is no tubulitis.

Patients with SjD and suspected kidney disease underwent kidney biopsy (KBx) as part of clinical care. As stated, we diagnosed a total of eight patients with SjD-TIN. In addition, five SjD-patients showed only features of SjD-CKD. Arterial hypertension and nephrocalcinosis were the main causes of CKD. As per definition, patients with SjD-TIN showed an interstitial inflammatory infiltrate. Figure 1a,b show the typical histopathology of SjD-TIN with a dense infiltrate of lymphocytes including T-cells, B-cells and plasma cells. Mononuclear lymphocytes can clearly be seen on the urinary side of the tubular basement membrane (tubulitis), having transmigrated from the renal interstitium (Fig. 1b, arrow). Figure 1c, d show histopathological images of a patient with SjD, who was biopsied for suspected TIN but showed only features of chronic kidney disease (SjD-CKD) such as interstitial fibrosis, tubular atrophy and, importantly, interstitial infiltration of leukocytes only into the fibrotic areas, without tubulitis.

Table 1 shows the BANFF classification for the SjD-TIN patients, confirming significant tubulitis in all of them. In line with reduced kidney function as per eGFR, all patients also showed a relevant degree of interstitial atrophy (ct) and fibrosis (ci). There was a trend towards more significant reduction in kidney function in patients with higher ct and ci [data not shown]. As expected, most biopsies of SjD-TIN patients were rich in plasma cells with no clear trend towards a dominant Ig-class. On the other hand, none of the SjD-CKD patients had tubulitis (t) or interstitial inflammation (i) as per BANFF-Classification [sup. table S1].

**Table 1:** Quantification of TIN by the mBANFF-Classification for SjD-TIN patients.

Patient	i	t	t-exact	ti	i-IFTA	ci	ct	pc
1	2	2	6	2	3	2	2	2
2	0	1	2	1	2	1	1	3
3	3	2	5	3	3	3	3	3
4	0	1	1	2	3	2	2	2
5	2	2	8	2	3	2	2	3
6	1	1	2	1	3	1	1	3
7	3	1	2	1	3	3	3	2
8	2	1	2	2	2	1	1	3
Median (range)	2 (0–3)	1 (1–2)	2 (1–8)	2 (1–3)	3 (2–3)	2 (1–3)	2 (1–3)	3 (2–3)

All kidney biopsies of patients with SjD-TIN were scored according to the modified BANFF-Classification (mBANFF)^25^. Percentage of non-scarred renal cortex area affected by inflammation (i), tubulitis score (t), number of mononuclear cells per 10 tubular epithelial cells (t-exact), percentage of total renal cortex area, including areas of interstitial fibrosis and tubular atrophy (IFTA) affected by interstitial inflammation (ti), percentage of scarred renal cortex area affected by inflammation (i-IFTA), percentage of interstitial area affected by fibrosis (ci), percentage of interstitial area with tubular atrophy (ct), percentage of plasma cells among the inflammatory cells (pc).

### Clinical parameters don’t differentiate between SjD-TIN and SjD-CKD

Table 2 shows the clinical characteristics of our SjD-TIN and SjD-CKD patient collectives as a whole. Supplementary tables S2 and S3 show clinical characteristics of each individual patient of both collectives. All patients were on stable immunosuppressive medication at the time of biopsy. Of the patients with biopsy proven SjD-TIN (n = 8) 87,5% were female, all were positive for SSA-antibodies and 75% were positive for SSB-antibodies. The median age at diagnosis of SjD-TIN was 46 years and the median disease duration was 2 years with a range of 0 to 12 years. None of the SjD-TIN patients had recently taken proton pump inhibitors (PPI), non-steroidal anti-inflammatory drugs (NSAID) or antibiotics, all of which are known to cause drug induced TIN. There was no difference in the prevalence of hypertension or diabetes mellitus between the groups. The two groups were also similar with regards to immunosuppressive medication (Table 2).

**Table 2:** Clinical parameters of SjD patients.

Characteristic	SjD-TIN	SjD-CKD
Female (absolute, %)	7/8 (87.5%)	4/5 (80%)
White (absolute, %)	7/8 (87.5%)	5/5 (100%)
Age – yr (median, IQR)	46 (28–74)	48 (34–70)
Classified as SjD (absolute, %)	8/8 (100%)	5/5 (100%)
Disease duration – yr (median, IQR)	2 (0–12)	2 (0–8.5)
Immunosuppressive medication other than HCQ at time of biopsy (absolute, %)	3/8 (37.5%)	2/5 (40%)
ESSDAI at diagnosis (median, range)	3 (0–7)	3 (0–9)
Renal ESSDAI pre-biopsy (median, range)	10 (0–15)	10 (0–15)
DM (absolute, %)	0/8 (0%)	0/5 (0%)
aHTN (absolute, %)	3/8 (37.5%)	2/5 (40%)
Other rheumatic diseases (absolute, %)	1/8 (12.5%)	1/5 (20%)
Abx/PPI/NSAID use prior to biopsy (absolute, %)	0/8 (0%)	2/5 (40%)
Serum creatinine – mg/dl (mean, SD)	1.7 (0.7)	1.4 (0.5)
BUN – mg/dl (mean, SD)	21.1 (9.0)	25.1 (14.7)
eGFR (CKD-EPI) – ml/min/1.73m^2^ (mean, SD)	44.4 (23.1)	53 (21.4)
uPCR – mg/mg (mean, SD)	983 (1122)	453 (640)
uACR – mg/mg (mean, SD)	353.0 (784.6)	82.2 (67.6)
Urinary A1-microglobulin to creatinine ratio mg/g	120.8 (127.6)	109.1 (165.9)
Dipstick protein > trace (absolute, %)	4/7 (57%)	2/5 (40%)
Dipstick leukocytes > trace (absolute, %)	3/7 (43%)	3/5 (60%)
Leukocytes in urinary sediment (absolute, %)	5/7 (71%)	4/5 (80%)
Dipstick Hb > trace (absolute, %)	1/7 (14%)	1/5 (20%)
C-reactive protein – mg/l (mean/SD)	1.9 (3.6)	6.1 (11)
Low K^+^ or K^+^ substitution needed (absolute, %)	2/8 (25%)	0/5 (0%)
C3 – mg/dl (mean, SD)	91.4 (28.7)	102.8 (15.3)
C3 reduced (absolute, %)	2/7 (28.6%)	1/5 (20%)
C4 – mg/dl (mean, SD)	22.8 (9.5)	25.8 (8.5)
C4 reduced (absolute, %)	2/7 (28.6%)	0/5 (0%)
Rheumatoid factor – IU/ml (mean, SD)	60.6 (34.5)	31.5 (26.2)
Anti-SSA-positive (absolute, %)	8/8 (100%)	4/4 (100%)
Anti-SSB-positive (absolute, %)	6/8 (75%)	2/3 (66.7%)

Relevant clinical parameters of SjD-TIN and SjD-CKD patients are tabulated. Classification of SjD according to the 2016 ACR/EULAR criteria^23^. Activity according to the EULAR Sjögren’s Syndrome Disease Activity Index (ESSDAI)^24^. Diabetes mellitus (DM). Arterial Hypertension (aHTN). Antibiotics (Abx). Proton pump inhibitors (PPI). Non-steroidal anti-inflammatory drugs (NSAID). Blood urea nitrogen (BUN). Estimated glomerular filtration rate (eGFR) according to Chronic Kidney Disease Epidemiology Collaboration (CKD-EPI). Urinary protein to creatinine ratio (uPCR). Urinary albumin to creatinine ratio (uACR). Complement C3 (C3). Complement C4 (C4). Sjögren’s-syndrome-related antigen A/B autoantibodies (SSA/SSB).

At the time of SjD-TIN diagnosis, patients had varying degrees of SjD activity with non-renal ESSDAI ranging from 0 to 7 with a median of 3. Serologic activity (increased IgG or decreased complement) and haematologic abnormalities (cytopenias) were the most common non-renal manifestations. These parameters were not different compared to patients with SjD-CKD (n = 5).

Next, we specifically looked at the renal aspect of the ESSDAI pre-biopsy (rESSDAI). This ranged from 0 to 15 with a median of 10 in both, SjD-TIN and SjD-CKD patients. Interestingly, leukocyturia on urine dipstick, a commonly used screening test for TIN, was similar in SjD-TIN and SjD-CKD patients with 43% and 40%, respectively. Urinary sediment was the more sensitive method to detect leukocyturia, but could also not differentiate SjD-TIN from SjD-CKD (71% versus 80%). Merely one patient in the SjD-TIN group indeed showed leukocyte casts. Similarly, only 57% of patients with SjD-TIN had proteinuria higher than “trace” on dipstick testing, compared to 40% of SjD-CKD patients, indicating, that this is an insufficiently sensitive screening test. Likewise, uPCR and uACR could not differentiate between the groups [sup. fig. S1]. Urinary tract infections were excluded by culture.

Serum creatinine and eGFR were also similar between the groups, as was the high proportion of patients with significantly reduced excretory renal function (eGFR < 60 ml/min; CKD stage ≥ 3) (6/8 versus 3/5), indicating significant kidney damage in both collectives.

Taken together, none of the clinical parameters including ESSDAI, rESSDAI, uPCR, uACR, prior immunosuppression, hypertension or diabetes mellitus were able to differentiate SjD-TIN from SjD-CKD.

### Urinary T cells are a non-invasive biomarker for SjD-TIN

Even though many of our patients didn’t show significant leukocyturia as per urine dipstick test (Table 2), leukocyturia is generally agreed to be one of the clinical hallmarks of TIN. Indeed 71% of our SjD-TIN patients showed leukocytes in the urinary sediment (Table 2). We thus postulated, that a more sensitive and precise method for identifying and quantifying urinary leukocytes (uLeuk) might improve diagnosis and could potentially also yield novel insights into the pathophysiology of SjD-TIN.

We therefore developed a multicolor flow cytometry (FC) panel to quantify and characterize uLeuk. We were able to differentiate neutrophils (PMN), eosinophils (Eos), classical and alternative macrophages/monocytes (class & alt M/M), B-cells, NK-cells, CD3^+^ T cells, CD8^+^ cytotoxic T cells and CD4^+^ T_H_ cells. Please refer to supplementary figure S2 for the detailed gating strategy. We measured the frequencies of specific uLeuk populations relative to the total CD45^+^ uLeuk. For absolute numbers, we used a similar approach to what is used for quantification of proteinuria in spot-urine samples and normalized the total leukocyte count detected via FC to ml of urine volume and gram of urinary creatinine.

In addition to SjD-TIN and SjD-CKD patients, we also studied leukocyturia from non-SjD CKD patients, healthy volunteers (HC) and patients with bacterial cystitis (UTI), who are known to have clinically very apparent leukocyturia.

**Fig. 2:**
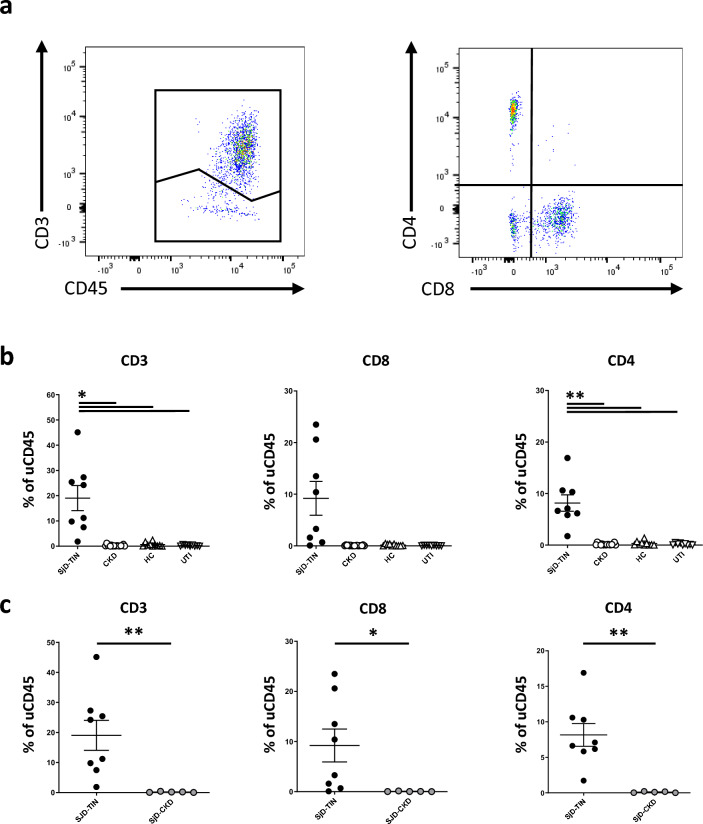
Quantification of relative T cells in the urine. (**a**) Representative flow cytometry plots of a patient with SjD-TIN. Pregating was performed as shown in sup. fig. S1. (**b**) Quantification of the indicated cell types in the indicated groups. CKD comprises patients with SjD-CKD (n = 5) and CKD patients without SjD (n = 12). HC comprises n = 10 healthy controls, UTI comprises n = 9 urinary tract infections. (**c**) Quantification of the indicated cell types in n = 8 SjD-TIN versus n = 5 SjD-CKD patients. Circles show individual patients, horizontal lines show mean values. Error bars show the standard error of the mean. ** p < 0.01, *p < 0.05.

We first looked at urinary T cell subtypes. Among these, pan-CD3^+^ T cells as well as CD8^+^ T cells and CD4^+^ T_H_ cells were clearly identified in the urine of SjD-TIN patients, as well as the control groups (Fig. 2a). Percentages of CD3^+^ T cells and CD4^+^ T_H_ cells but not CD8^+^ T cells were significantly increased in patients with SjD-TIN, compared to all other groups (Fig. 2b). Next, we specifically compared SjD-TIN and SjD-CKD, as these are the clinically most relevant groups, that need to be diagnostically distinguished. Percentages of CD3^+^ T cells, CD8^+^ T cells and CD4^+^ T_H_ cells were significantly increased in patients with SjD-TIN compared to SjD-CKD (Fig. 2c). Together these data indicate, that frequencies of urinary CD3^+^ T cells in general and in particular CD4^+^ T_H_ cells are able to differentiate between SjD-TIN and other forms of renal disease, including SjD-CKD and UTI. Importantly, using a cutoff of 1,54% (+/− 0.18) urinary CD4^+^ T_H_ cells could differentiate SjD-TIN from the control groups with 100% sensitivity and specificity (Fig. 2b,c).

We also evaluated absolute urinary leukocyte cell numbers. Compared to HC and CKD, SjD-TIN had significantly higher absolute CD3^+^, CD8^+^ as well as CD4^+^ T_H_ cell numbers [sup. fig. S3a]. UTIs on the other hand had very high absolute CD3^+^, CD8^+^ and CD4^+^ T_H_ cell numbers and showed no difference to SjD-TIN [sup. fig. S3a]. In direct comparison to SjD-CKD patients, SjD-TIN showed significantly higher absolute CD3^+^, CD8^+^ as well as CD4^+^ T_H_ numbers [sup. fig. S3b]. However, in contrast to relative frequencies, absolute leukocyte numbers showed a much higher overlap between the groups and no clear cutoff with high sensitivity and specificity for differentiation of SjD-TIN from the other groups could be defined.

Finally, we also investigated other uLeuk populations. While there were some differences, neither absolute numbers nor relative frequencies of PMN, M/M, Eos, NK cells and B cells reliably differentiated SjD-TIN from the control groups [sup. fig. S4a-d].

### Urinary T cells correlate strongly with histological activity

**Fig. 3:**
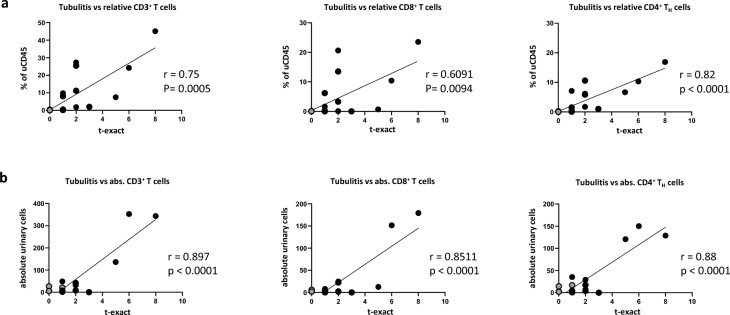
Correlation of histological severity of TIN with urinary T cells. (**a**) Correlation of the severity of tubulitis according to the invading mononuclear cells per 10 tubular epithelial cells (t-exact) with the relative urinary frequency of the indicated cell types. (**b**) Correlation of the severity of tubulitis according to the invading mononuclear cells per 10 tubular epithelial cells (t-exact) with the absolute urinary concentration of the indicated cell types. Black and grey circles show individual patients with SjD-TIN and SjD-CKD respectively. r is Pearson’s correlation coefficient. The line represents the simple linear regression. Absolute urinary cells are normalized to mg of urinary creatinine.

Next, we wanted to investigate, whether uLeuk would reflect the histological severity of TIN. We thus correlated absolute and relative urinary CD3^+^ T cells, CD8^+^ cytotoxic T cells and CD4^+^ T_H_ cells with the different aspects of the mBANFF-classification. Indeed, we observed a strong and significant correlation between the tubulitis score (t) and percentages of urinary CD3^+^ T cells, CD8^+^ cytotoxic T cells and CD4^+^ T_H_ cells, as well as absolute urinary CD3^+^ T cells and CD4^+^ T_H_ cells [sup. fig S5]. The other parameters of the mBANFF classification did not significantly correlate with uLeuks. For an even more precise characterization of the relationship between tubulitis and leukocyturia we next correlated the exact number of mononuclear cells per 10 tubular epithelial cells (t-exact) with urinary T cells. This analysis showed an even stronger correlation for CD3^+^ T cells, CD8^+^ cytotoxic T cells and CD4^+^ T_H_ cells, with CD4^+^ T_H_ cells being substantially better than CD8^+^ cytotoxic T cells (Fig. 3). In contrast, there was no correlation between histology or urinary CD4^+^ T_H_ cells with any of the clinical markers we examined.

### Urinary T cells are a non-invasive biomarker for treatment response of SjD-TIN

As another important goal, we wanted to study, whether urinary T cells could be a novel biomarker to assess response to treatment. All patients with SjD-TIN had their immunosuppression intensified after kidney biopsy. We defined two time points for urinalysis after initiation of treatment. One was the first follow-up in our outpatient department after initial change of therapy (first FU), which occurred at a median of 37 days. The other was the last follow-up before finalization of data collection (last FU), which occurred at a median of 360 days after biopsy. The prescribed immunosuppressive medication and the time to first- and last follow-up are shown in Table 3.

**Table 3:** Treatment and follow-up.

Patient	Treatment	Days to first follow-up	Days to last follow- up
1	Prednisolone	20	131
2	Rituximab	54	208
3	Prednisolone + Azathioprine	20	636
4	Prednisolone (+ Azathioprine at day 7)	7	252
5	Prednisolone (+ Mycophenolate mofetil at day 14)	13	636
6	Rituximab	62	468
7	Rituximab	134	813
8	Azathioprine	104	181
		Median: 37	Median: 360

This table shows the immunosuppressive treatment, that was initiated for the SjD-TIN patients. Prednisolone was initiated at approximately 1 mg/kg body weight. Azathioprine was given at a dose of 2 mg/kg body weight. The dose of mycophenolat mofetil was 2 g/day. Rituximab induction was initiated with two doses of 1 g (2 weeks apart) and maintenance was 1000 mg every 6 months as clinically indicated. First and last follow-up were the first and last time points when patients were seen in our outpatient clinic respectively with the days since initiation of therapy documented.

**Fig. 4:**
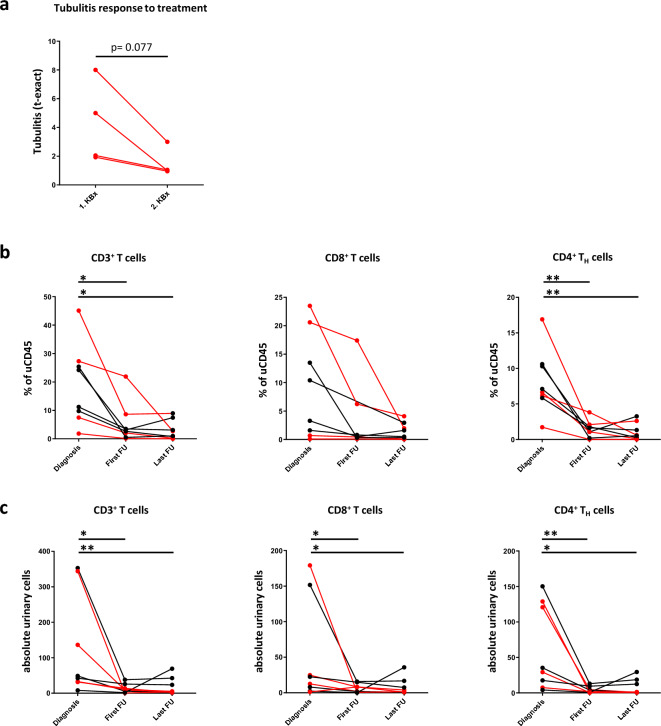
Response to treatment. (**a**) Change of tubulitis upon treatment (median of 549 days) of the 4 patients in whom follow up biopsies were performed. Tubulitis is shown according to the number of invading mononuclear cells per 10 tubular epithelial cells (t-exact). Change of the indicated (**b**) relative and (**c**) absolute urinary cell populations during treatment. All time points for all patients with SjD-TIN are plotted individually. The 4 patients with repeat biopsies are highlighted in red. Absolute urinary cells are normalized to mg of urinary creatinine. ** p < 0.01, *p < 0.05.

Repeat kidney biopsies of four SjD-TIN patients at a median of 549 days after treatment initiation showed a strong trend (p = 0.077) towards improvement in tubulitis, indicating clinical effects of the prescribed treatment on SjD-TIN-activity (Fig. 4a). Clinical parameters at first FU and last FU also indicated improvement of systemic SjD activity. In particular, total IgG as an important serological marker showed a significant reduction at the last FU [sup. fig. S6a], while Rheumatoid factor and complement did not change over time [sup. fig. S6b].

In terms of renal parameters, uPCR fell in most patients [sup. fig. S6c], but did not reach the required level of statistical significance at the last FU. Renal function, as measured by serum creatinine and eGFR, did not deteriorate further [sup. fig. S6d]. Both parameters thus indicated stabilization or improvement of renal disease, albeit with low sensitivity and poor diagnostic power.

Importantly, however, treatment of SjD-TIN led to a quick and sustained reduction in relative and absolute urinary T-lymphocytes (Fig. 4b,c, patients with repeat biopsies as shown in Fig. 4a are indicated by red lines). Compared to the time point when active TIN was first diagnosed by biopsy, relative and absolute CD3^+^ T cell numbers fell significantly both at the first FU as well as the last FU. Absolute but not relative CD8^+^ T cell numbers also fell significantly. The strongest alterations, however, were once again found with CD4^+^ T_H_ cells, whose relative and absolute numbers fell significantly at both FU time points.

Concerning other urinary leukocyte populations, there was comparatively little change during treatment. Only absolute numbers, but not relative frequencies of NK cells and B cells were lower at either first FU or last FU respectively. M/M and Eos showed no significant change and relative PMN were even higher at the last FU [sup. fig. S7].

### Rise in urinary CD4^+^ T_H_ cells can predict development of SjD-TIN

**Fig. 5:**
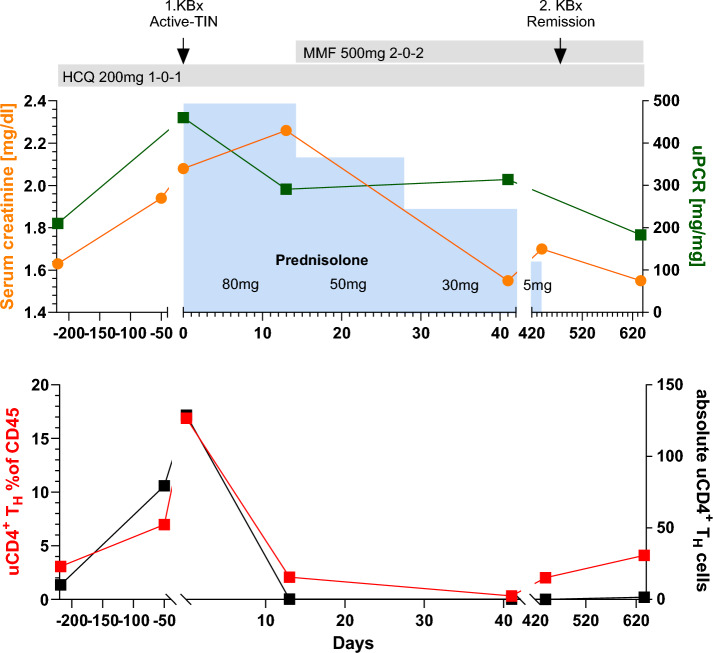
Detailed analysis of uCD4^+^ T_H_ cells during the clinical course of patient 5. The top graph shows changes in serum creatinine and proteinuria (in mg protein per mg of urinary creatinine) from 218d before the first biopsy, until 620d thereafter. Time points of kidney biopsies (KBx) and treatment is shown at the top (HCQ: hydroxychloroquine, MMF: mycophenolate mofetil). The bottom graph shows the change in relative and absolute urinary CD4^+^ T_H_ cells over the same time. Absolute urinary cells are normalized to mg of urinary creatinine.

For one of our SjD-TIN patients we had longitudinal leukocyturia data from 218 days prior to biopsy proven TIN (Fig. 5). Compared to the earliest time point (-218d), we saw a strong increase in urinary CD4^+^ T_H_ cells, at the time of biopsy (0d). Upon treatment, we documented a marked fall in absolute and relative urinary CD4^+^ T_H_ cells, in parallel with improvement of clinical parameters (uPCR, serum creatinine). In line with reduced urinary CD4^+^ T_H_ cells, a repeat kidney biopsy at day 480 showed a significant reduction of tubulitis (BANFF t from 2 to 1 and t-exact from 8 to 2).

## Discussion

There is currently no validated biomarker to help with diagnosis or assessment of therapeutic response regarding SjD-TIN. While the renal domain of the ESSDAI comprises various non-invasive markers like proteinuria, eGFR, haematuria and tubular acidosis^24^, none of these are specific for SjD associated kidney involvement and don´t allow the diagnosis of SjD-TIN. In particular, proteinuria, dipstick testing and urinary sediment are routinely done in clinical practice. Our data, however, clearly show the limitations of these non-invasive tests.

In search of novel and more suitable markers, we here report that urinary T lymphocytes in general and CD4^+^ T_H_ cells in particular are increased in SjD-TIN patients. Especially the relative frequencies of CD4^+^ T_H_ cells could differentiate between SjD-TIN and SjD-CKD, as well as HC and UTIs with a very good sensitivity and specificity. In fact, the lowest SjD-TIN patient had 1.7% CD4^+^ T_H_ cells and the highest control had 1.4%. Therefore, a cut-off between these two values would allow stringent differentiation between SjD-TIN and the controls in our dataset. Urinary CD4^+^ T_H_ cells are thus a potential new biomarker for diagnosis of SjD-TIN.

We could further show, that relative urinary T-cells correlate very well with histological severity of tubulitis in SjD patients. This further implies their potential usefulness as a biomarker and gives credence to our immunopathological understanding of TIN, which links tubulitis to leukocyturia and kidney injury.

Furthermore, urinary CD4^+^ T_H_ cells fell upon treatment of SjD-TIN, both in relative and absolute terms. It is of note, that T cell counts correlated well with histological findings in four of our SjD-TIN patients with repeat biopsies. This indicates, that they are also a potential biomarker for monitoring treatment response. From a clinical point of view, this aspect is particularly interesting, since it might help to avoid otherwise necessary repeat kidney biopsies, which bear the risk of potential complications.

TIN in SjD is often described as a relatively benign condition. However, our cohort had a significant reduction in kidney function with a mean eGFR of 44 ml/min/1.73 m^2^ at the time of biopsy and this did not significantly improve upon treatment. This level of chronic kidney disease is associated with a high risk for cardiovascular disease and CKD progression. The significant degree of reduced kidney function prior to diagnosis shows, that irreversible kidney damage had already occurred bevor SjD-TIN became clinically apparent. This further signifies the need for more sensitive biomarkers like urinary CD4^+^ T_H_ cells, which might be used as a non-invasive screening test for SjD-TIN.

Recommendations by EULAR and others regarding treatment of SjD-TIN exist, but strong evidence is lacking^27^. We believe that better phenotyping of SjD-TIN and the use of sensitive biomarkers for early diagnosis and follow up, such as urinary CD4^+^ T_H_ cells, might help to refine treatment modalities and improve the outcome.

A clear limitation of our study is the small sample size with only eight SjD-TIN and 5 SjD-CKD patients. Clinical visits and repeat biopsies were only done, when clinically indicated, potentially leading to skewing of our data. Our study was also conducted in a single center and needs to be corroborated with data from other independent cohorts.

Taken together, our results point towards urinary CD4^+^ T_H_ cells as a useful non-invasive biomarker for diagnosis and follow-up of SjD-TIN, which might help to improve early diagnosis and clinical outcome.

## Supplementary Information

Supplementary Information.

## Data Availability

All data supporting the findings of this study are available within the paper and its Supplementary Information.
