# Supplementary material for: Urinary CD4+ T helper cells are a potential biomarker for tubulointerstitial nephritis in Sjögren’s disease
Source: Sci Rep. 2026 Feb 11;16:5943. doi: 10.1038/s41598-025-34685-x (PMC12894895; doi:10.1038/s41598-025-34685-x)
Supplement: Supplementary file 1 — Supplementary Information. [file 41598_2025_34685_MOESM1_ESM.pdf]

Supplementary Table S1. Quantification of TIN by mBANFF-Classification for SjD-CKD patients								
**Patient**	**i**	**t**	**t-exact**	**ti**	**i-IFTA**	**ci**	**ct**	**pc**
9	0	0	0	0	1	2	2	1
10	0	0	1	0	0	1	1	0
11	No Data	No Data	No Data	No Data	No Data	No Data	No Data	No Data
12	0	0	0	1	1	1	1	0
13	0	0	0	0	1	2	2	0
Median (range)	0 (0)	0 (0)	0 (0-1)	0 (0-1)	1 (0-1)	1,5 (1-2)	1,5 (1-2)	0 (0-1)

Supplementary table S1. Quantification of TIN by the mBANFF-Classification for SjD-CKD patients

Five kidney biopsies of patients with SjD-CKD were scored according to the mBANFF-Classification [25]. Percentage of non-scarred renal cortex area affected by inflammation (i), tubulitis score (t), number of mononuclear cells per 10 tubular epithelial cells (t-exact), percentage of total renal cortex area, including areas of interstitial fibrosis and tubular atrophy (IFTA) affected by interstitial inflammation (ti), percentage of scarred renal cortex area affected by inflammation (i-IFTA), percentage of interstitial area affected by fibrosis (ci), percentage of interstitial area with tubular atrophy (ct), percentage of plasma cells among the inflammatory cells (pc).

Supplementary Table S2. Detailed clinical characteristics of SjD-TIN patients

**Patient**	**1**	**2**	**3**	**4**	**5**	**6**	**7**	**8**
Sex	female	female	female	female	male	female	female	female
Race	white	white	white	white	white	white	black	white
Age – yr	43	74	60	49	59	28	36	43
Classified as SjD	yes	yes	yes	yes	yes	yes	yes	yes
Disease duration – yr	7	12	0	0	3	2	0,5	2
Immunosuppression at time of Biopsy	Hydroxychloroquine	Prednisolone 10mg	none	none	Hydroxychloroquine	Mycophenolate mofetil	Mycophenolate mofetil	Hydroxychloroquine
ESSDAI at diagnosis (without renal)	3	7	0	1	4	3	6	2
ESSDAI at diagnosis (without renal) with domains	Haematological: 2<br>Biological: 1	Articular: 2<br>Haematological: 4<br>Biological: 1		Biological: 1	Haematological: 2<br>Biological: 2	Haematological: 2<br>Biological: 1	Haematological: 4<br>Biological: 2	Articular: 2
Renal ESDAI at diagnosis (pre-biopsy)	10	10	10	15	10	5	10	0
DM	no	no	no	no	no	no	no	no
aHTN	no	no	no	yes	yes	no	no	yes
Other rheumatic disease	no	yes (lcSSC)	no	no	no	no	no	no
Abx/PPI/NSAID use prior to IN Diagnosis	no	no	no	no	no	no	no	no
Serum creatinine – mg/dl	1,73	1,09	3,05	2,28	2,08	0,88	1,51	1,12
BUN – mg/dl	15,40	16,6	Not recorded	31,5	17,8	10,3	21,1	35
eGFR (CKD-EPI) – ml/min/1.73m^2	38	47	16	22	34	114	42	61
uPCR – mg/mg	1655	562	1597	3282	460	201	158	0
uACR – mg/mg	Not recorded	14	218	2124	14	0	72	29
Urinary A1-microglobulin to creatinine ratio mg/g	250,9	76,5	306,1	79,3	Not recorded	12,1	Not recorded	0
Dipstick protein > trace	Not recorded	no	Yes	yes	yes	yes	no	no
Dipstick leucocytes > trace	Not recorded	no	Yes	yes	no	no	no	yes
Leukocytes in urinary sediment	yes	no data	yes	yes	no	yes	no	yes
Dipstick Hb > trace	Not recorded	no	No	yes	no	no	no	no
C reactive protein – mg/l	0	6	9	0	0	0	0	0
Low K^+ or K^+ substitution needed	yes	no	no	no	no	yes	no	no
C3 – mg/dl	88	105	Not recorded	93	30	118	103	103
C4 – mg/dl	19,3	11,8	Not recorded	40,3	< 1	20,1	23,5	21,8
Rheumatoid factor – IU/ml	57,1	43,5	18,5	0	1148,9	59,8	36,2	85
Anti-SSA-positive	> 240	> 240	> 240	94	> 240	> 240	positive	> 240
Anti-SSB-positive	60	> 320	19	<0,4	< 0,4	339	positive	139
ANA-Titer	1:5120	positive	1:1280	1:160	1:5120	> 1:5120	positive	1:5120
Medication before KBx	– Hydroxychloroquine<br>– Paroxetine<br>– L-Thyroxine<br>– Sodium bicarbonate<br>– Vitamin D<br>– Potassium	– Letrozole<br>– Trastuzumab<br>– Vitamin D<br>– Prednisolone 10mg		– Ramipril	– Hydroxychloroquine<br>– Bisoprolol<br>– Ramipril	– Mycophenolate mofetil<br>– Vitamin D<br>– Potassium<br>– Birth control pill	– Mycophenolate mofetil<br>– Folic acid<br>– Vitamin D	– Hydroxychloroquine<br>– Candesartan<br>– Vitamin D<br>– Metamizole<br>– Tramadol<br>– Birth control pill
Changes to Immunosuppression after KBx	– Prednisolone 50 mg<br>– Mycophenolate mofetil	– Rituximab 1000 mg (d1 and d15)	– Prednisolone 60 mg<br>– Azathioprine	– Prednisolone 60 mg<br>– Azathioprine 50 mg	– Prednisolone 80 mg<br>– Mycophenolate mofetil	– Rituximab 1000 mg (d1 and d15)<br>– Rituximab 500 mg (after 6 months) 01/24<br>– Mycophenolat mofetil stopped	– Rituximab 1000 mg (d1 and d15)<br>– Mycophenolat mofetil stopped	– Azathioprine

Supplementary table S2. Detailed clinical characteristics of SjD-TIN patients

This table is an extension of table 2 and shows detailed clinical parameters for all SjD-TIN patients. Activity according to the EULAR Sjögren's Syndrome Disease Activity Index (ESSDAI). Diabetes mellitus (DM). Arterial Hypertension (aHTN). Antibiotics (Abx). Proton pump inhibitors (PPI). Non-steroidal anti-inflammatory drugs (NSAID). Blood urea nitrogen (BUN). Estimated glomerular filtration rate (eGFR) according to Chronic Kidney Disease Epidemiology Collaboration (CKD-EPI). Urinary protein to creatinine ratio (uPCR). Urinary albumin to creatinine ratio (uACR). Complement C3 (C3). Complement C4 (C4). Sjögren's-syndrome-related antigen A/B autoantibodies (SSA/SSB).

Supplementary Table S3. Detailed clinical characteristics of SjD-CKD patients					
**Patient**	**9**	**10**	**11**	**12**	**13**
Sex	male	female	female	female	female
Race	white	white	white	white	white
Age – yr	61	70	39	34	48
Classified as SjD	yes	yes	yes	yes	yes
Disease duration – yr	0	8,5	2	7	0
Immunosuppression at time of Biopsy	None	Prednisolone 5mg<br>Abatacept	Azathioprine	None	None
ESSDAI at diagnosis (without renal)	9	0	4	1	3
ESSDAI at diagnosis (without renal) with domains	Articular: 4<br>Pulmonary: 5	none	Heamatological:2<br>Biological: 2	Biological: 2	Heamatological: 2<br>Biological: 1
Renal ESDAI at diagnosis (pre-biopsy)	10	15	5	5	15
DM	no	no	no	no	no
aHTN	no	yes	no	no	yes
Other Rheumatic Disease	yes (suspected SSc)	no	no	no	no
Abx/PPI/NSAID use prior to IN Diagnosis	no	yes (NSAR)	yes (PPI)	no	no
Serum creatinine – mg/dl	1,38	1,51	0,96	0,94	2,13
BUN – mg/dl	17,2	27,5	12,5	18,6	49,6
eGFR (CKD-EPI) – ml/min/1.73m^2	55,68	36,21	67	79	27
uPCR – mg/mg	442	1561	133	0	130
uACR – mg/mg	165	124	0	30	92
Urinary A1-microglobulin to creatinine ratio mg/g	27,2	300	<69,9	Not recorded	Not recorded
Dipstick protein > trace	yes	no	no	no	yes
Dipstick leucocytes > trace	no	no	yes	yes	yes
Leukocytes in urinary sediment	yes	yes	yes	yes	no
Dipstick Hb > trace	150	10	0	0	0
C reactive protein – mg/l	5,3	25,4	<4	<4	<5
Low K^+ or K^+ substitution needed	no	no	no	no	no
C3 – mg/dl	126	110	97	89	92
C4 – mg/dl	40	23	23,7	25,2	17
Rheumatoid factor (IgG) – IU/ml	50	0,6	12,4	12,9	Not recorded
Anti-SSA-positive	positive	>240	>240	>240	positive
Anti-SSB-positive	negative	0,4	48	212	Not recorded
ANA-Titer	1:320	>1:5120	>1:5120	>1:5120	positive
Most likely underlying disease for CKD according to clinical parameters and histopathology.	Uncharacteristic, possible aHTN	aHTN, IgA-Nephropathy	Nephrocalcinosis	Nephrocalcinosis	aHTN

Supplementary table S3. Detailed clinical characteristics of SjD-CKD patients

This table is an extension of table 2 and shows detailed clinical parameters for all SjD-CKD patients. Activity according to the EULAR Sjögren's Syndrome Disease Activity Index (ESSDAI). Diabetes mellitus (DM). Arterial Hypertension (aHTN). Antibiotics (Abx). Proton pump inhibitors (PPI). Non-steroidal anti-inflammatory drugs (NSAID). Blood urea nitrogen (BUN). Estimated glomerular filtration rate (eGFR) according to Chronic Kidney Disease Epidemiology Collaboration (CKD-EPI). Urinary protein to creatinine ratio (uPCR). Urinary albumin to creatinine ratio (uACR). Complement C3 (C3). Complement C4 (C4). Sjögren's-syndrome-related antigen A/B autoantibodies (SSA/SSB).

Supplementary Figure S1. Clinical parameters in SjD-TIN and SjD-CKD patients

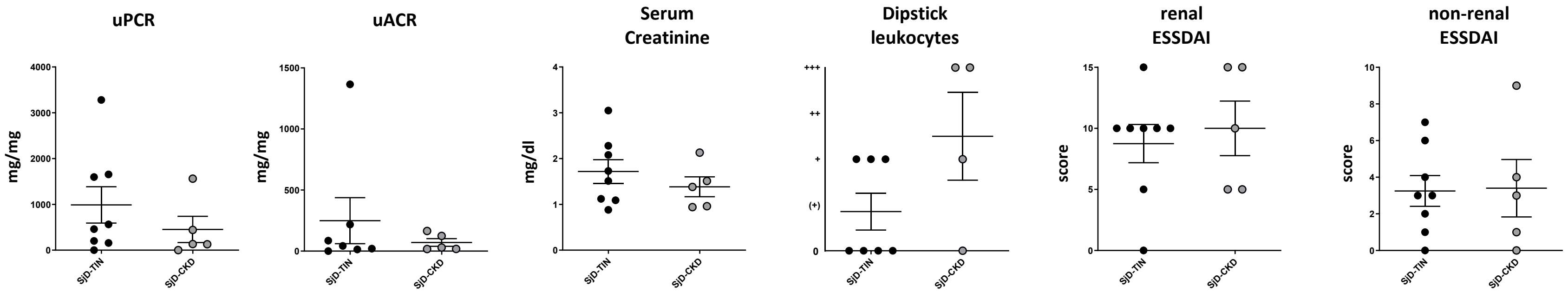

<u>Supplementary figure S1. Clinical parameters in SjD-TIN and SjD-CKD patients</u>

Comparison of the indicated clinical parameters between SjD-TIN and SjD-CKD patients at the time of biopsy. Urinary protein to creatinine ratio (uPCR). Urinary albumin to creatinine ratio (uACR). Serum creatinine (sCrea). Activity according to the EULAR Sjögren's Syndrome Disease Activity Index (ESSDAI). Circles show individual patients, horizontal lines show mean values. Error bars show the standard error of the mean.

Supplementary Figure S2. Detailed gating strategy

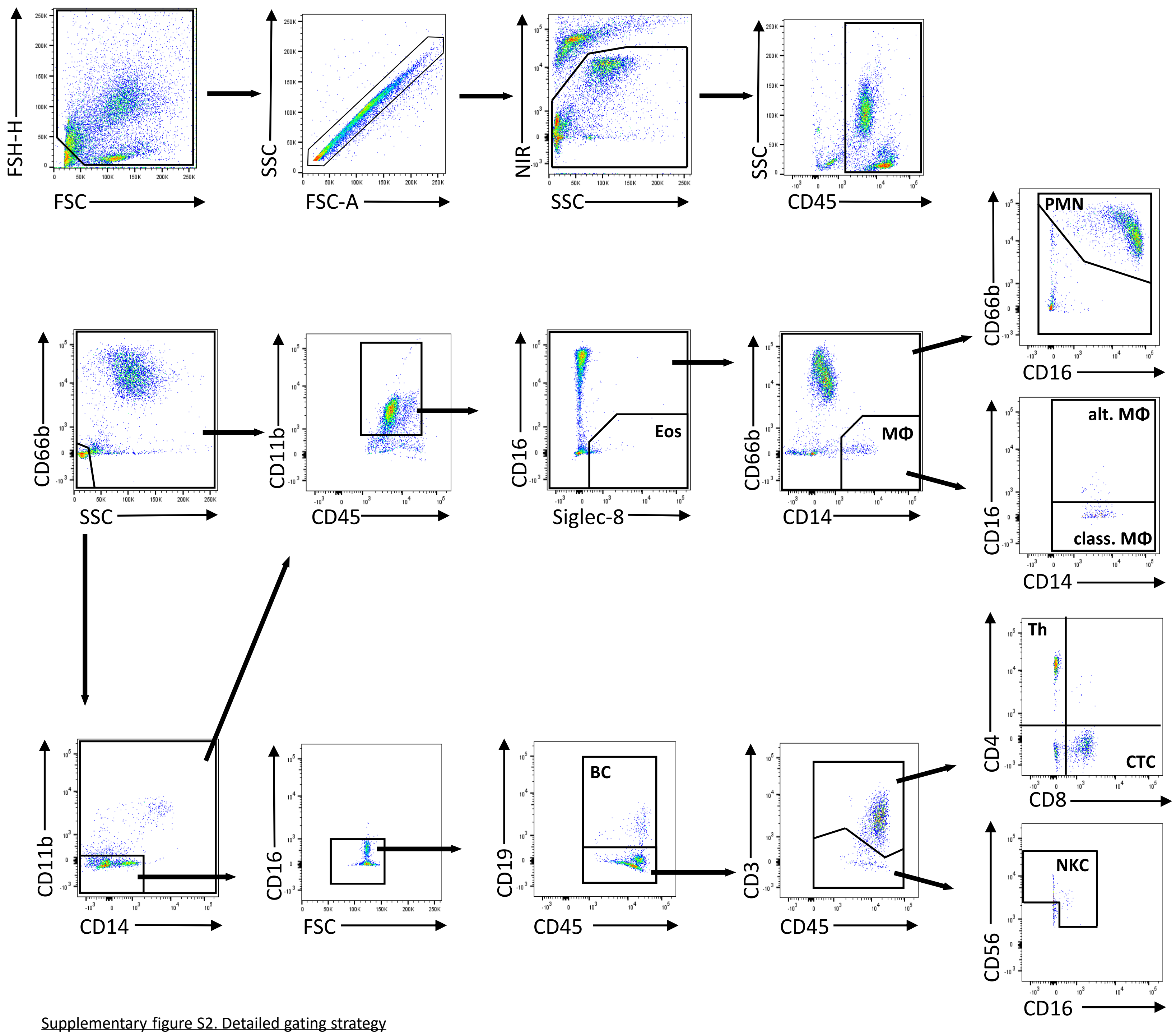

Supplementary figure S2. Detailed gating strategy

Urinary leukocytes were gated according to the demonstrated strategy. Initial gating was for live, single cells that are CD45 positive. Lymphocytes were defined as CD66b negative, SSC low, CD11b negative and CD16 negative/low. CD19 positive cells were defined as B cells. CD19 negative but CD3 positive cells were defined as T cells. These were further divided into CD4 positive and CD8 positive cells. The CD3 negative cells were classified as NK cells if they were CD56 positive and/or CD16 positive. CD66b positive and/or SSC int/high together with the CD11b positive and/or CD14 positive fraction of the CD66b negative, SSC low cells were used as the basis for myelocyte gating. Myelocytes were further defined as CD11b positive. Siglec-8 positive and CD16 negative cells were defined as eosinophils. The remaining cells were further gated and the CD66b negative and CD14 positive fraction was defined as macrophages/monocytes (M/M). CD16 was used to define classical M/M (CD16 negative) and alternative M/M (CD16 positive). Finally, of the remaining myelocytes, CD66b and CD16 double positive cells were defined as neutrophils.

Supplementary Figure S3. Quantification of absolute T cells in the urine

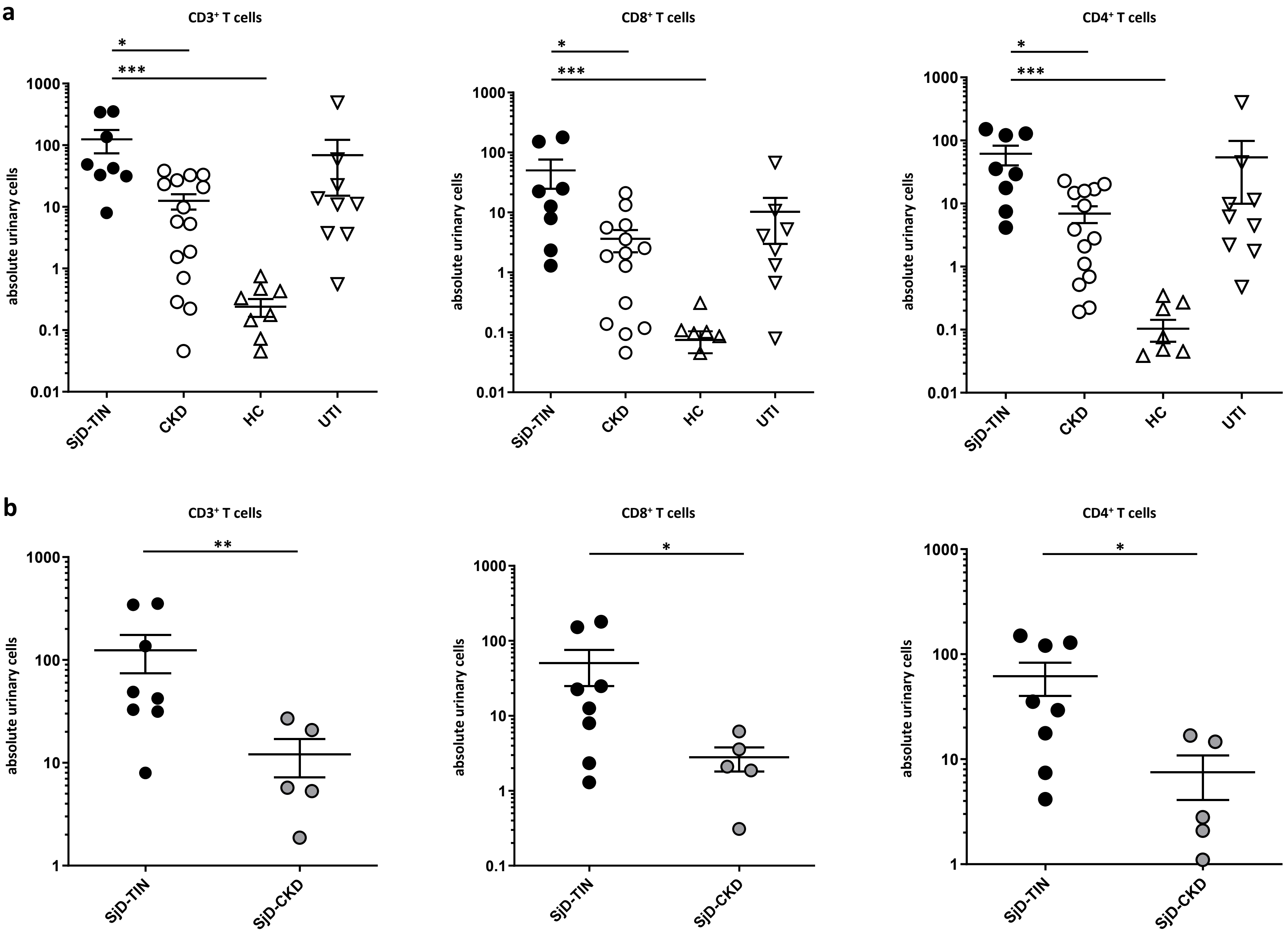

Supplementary figure S3. Quantification of absolute T cells in the urine

(a) Quantification of the indicated T cell types in the indicated patient groups. CKD comprises patients with SjD-CKD (n=5) and patients without SjD (n=12). (b) Quantification of the indicated cell types in the clinically most relevant groups (SjD-TIN and SjD-CKD). Circles show individual patients, horizontal lines show mean values. Error bars show the standard error of the mean. Absolute urinary cells are normalized to mg of urinary creatinine. *** $p<0.001$, ** $p<0.01$, *$p<0.05$.

Supplementary Figure S4. Quantification of relative and absolute non-T cell urinary leukocyte populations

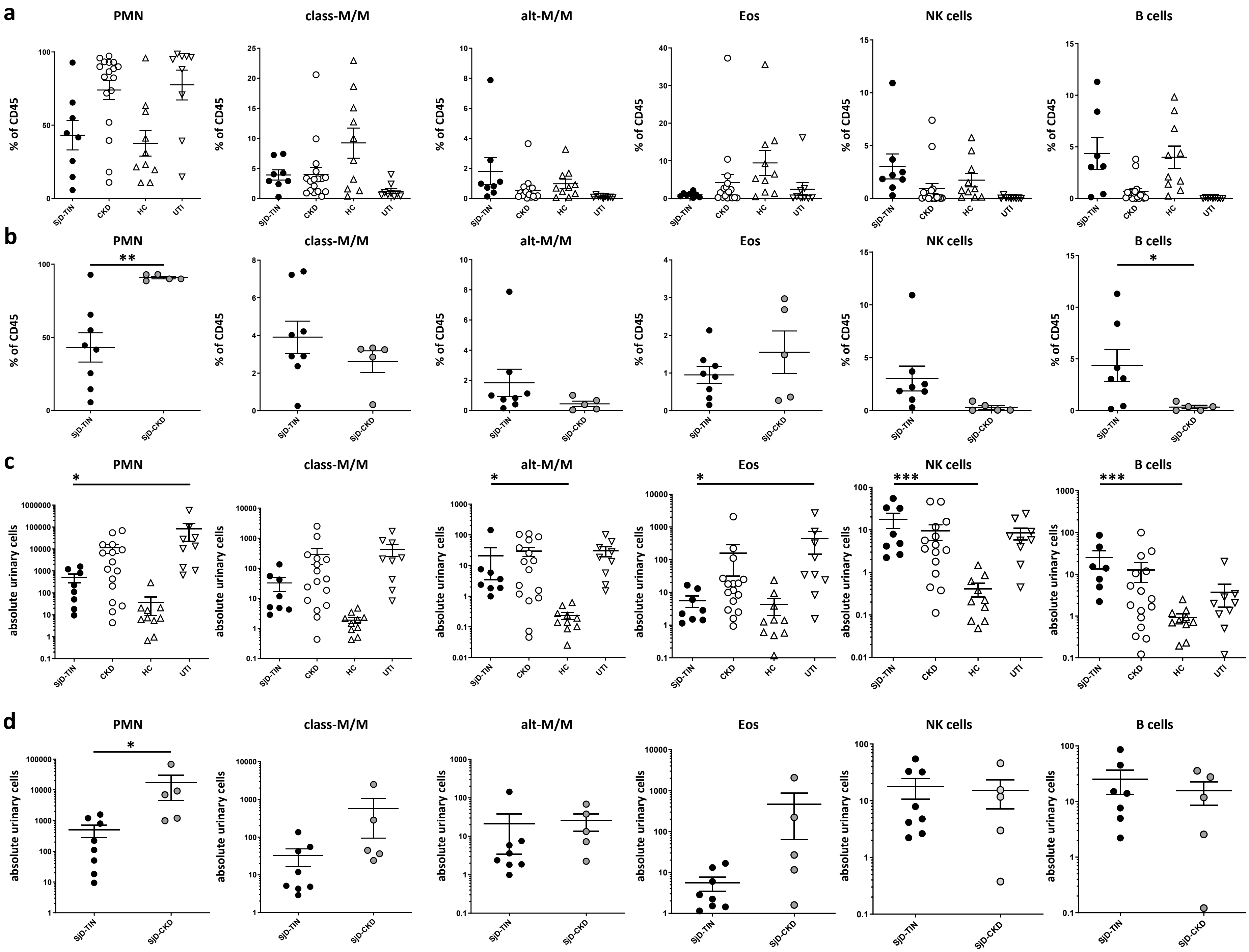

Supplementary figure S4. Quantification of relative and absolute non-T cell urinary leukocyte populations

(a) Quantification of urinary cell numbers as percentages of CD45 positive leukocytes of the indicated cell types in the indicated patient groups. CKD comprises patients with SjD-CKD (n=5) and patients without SjD (n=12). (b) Quantification of relative cell numbers of the indicated cell types in the clinically most relevant patient groups (SjD-TIN and SjD-CKD). Circles show individual patients, horizontal lines show mean values. (c) Quantification of absolute urinary cell numbers of the indicated cell types in the indicated patient groups. CKD comprises patients with SjD-CKD (n=5) and patients without SjD (n=12). (d) Quantification of absolute cell numbers of the indicated cell types in the clinically most relevant groups (SjD-TIN and SjD-TIN). Circles show individual patients, horizontal lines show mean values. Error bars show the standard error of the mean. Absolute urinary cells are normalized to mg of urinary creatinine. ** p<0.01, *p<0.05.

Supplementary Figure S5. Correlation of histological severity of TIN with urinary T cells

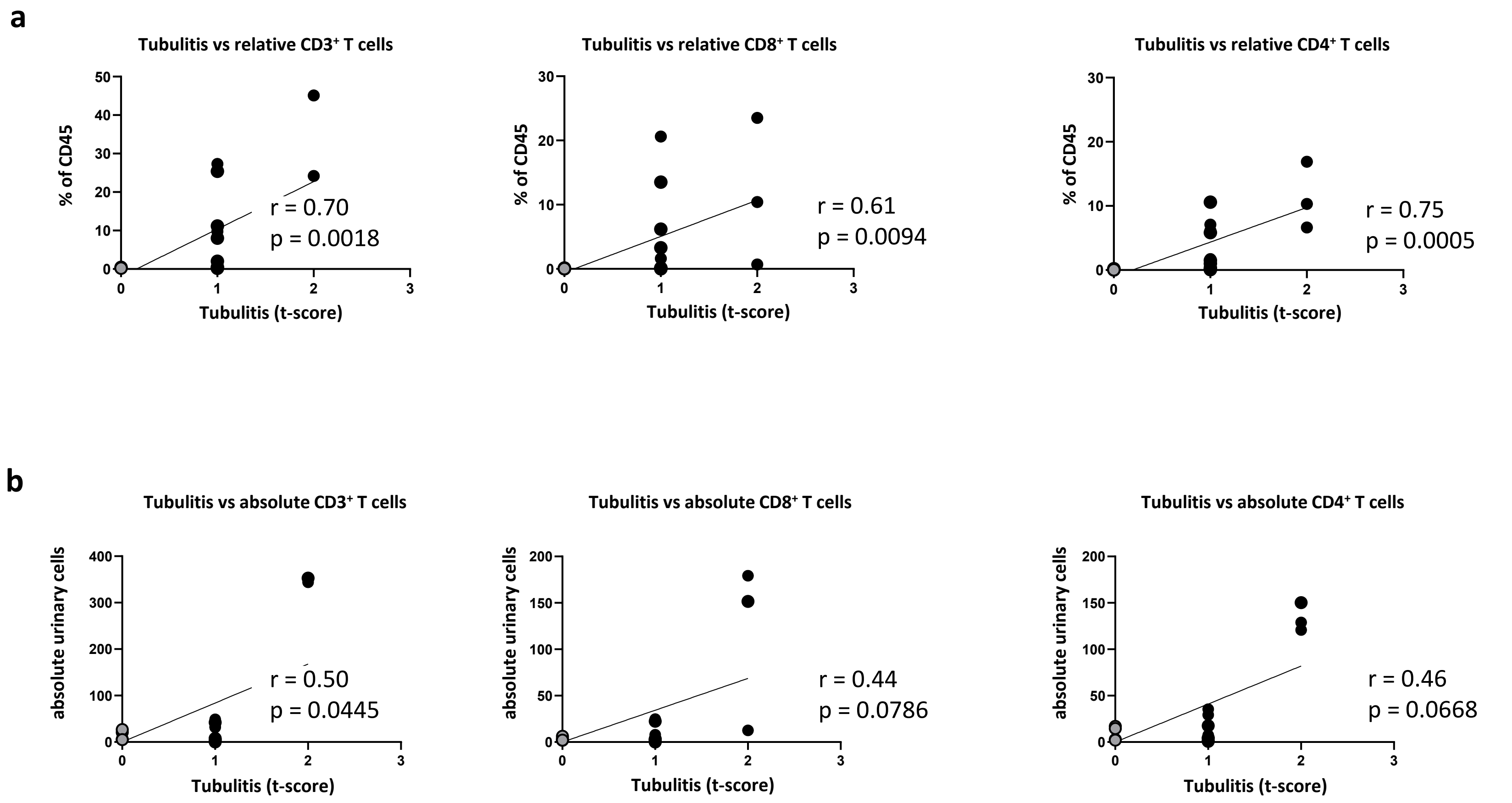

Supplementary figure S5. Correlation of histological severity of TIN with urinary T cells

(a) Correlation of the severity of tubulitis according to the mBANFF classification (t-score) with the relative urinary frequency of the indicated cell types. (b) Correlation of the severity of tubulitis according to the mBANFF classification (t-score) and the absolute urinary numbers of the indicated cell types. Black and grey circles show individual patients with SjD-TIN and SjD-CKD respectively. r is Spearman’s correlation coefficient. The line represents the simple linear regression. Absolute urinary cells are normalized to mg of urinary creatinine.

Supplementary Figure S6. Response of clinical parameters to treatment

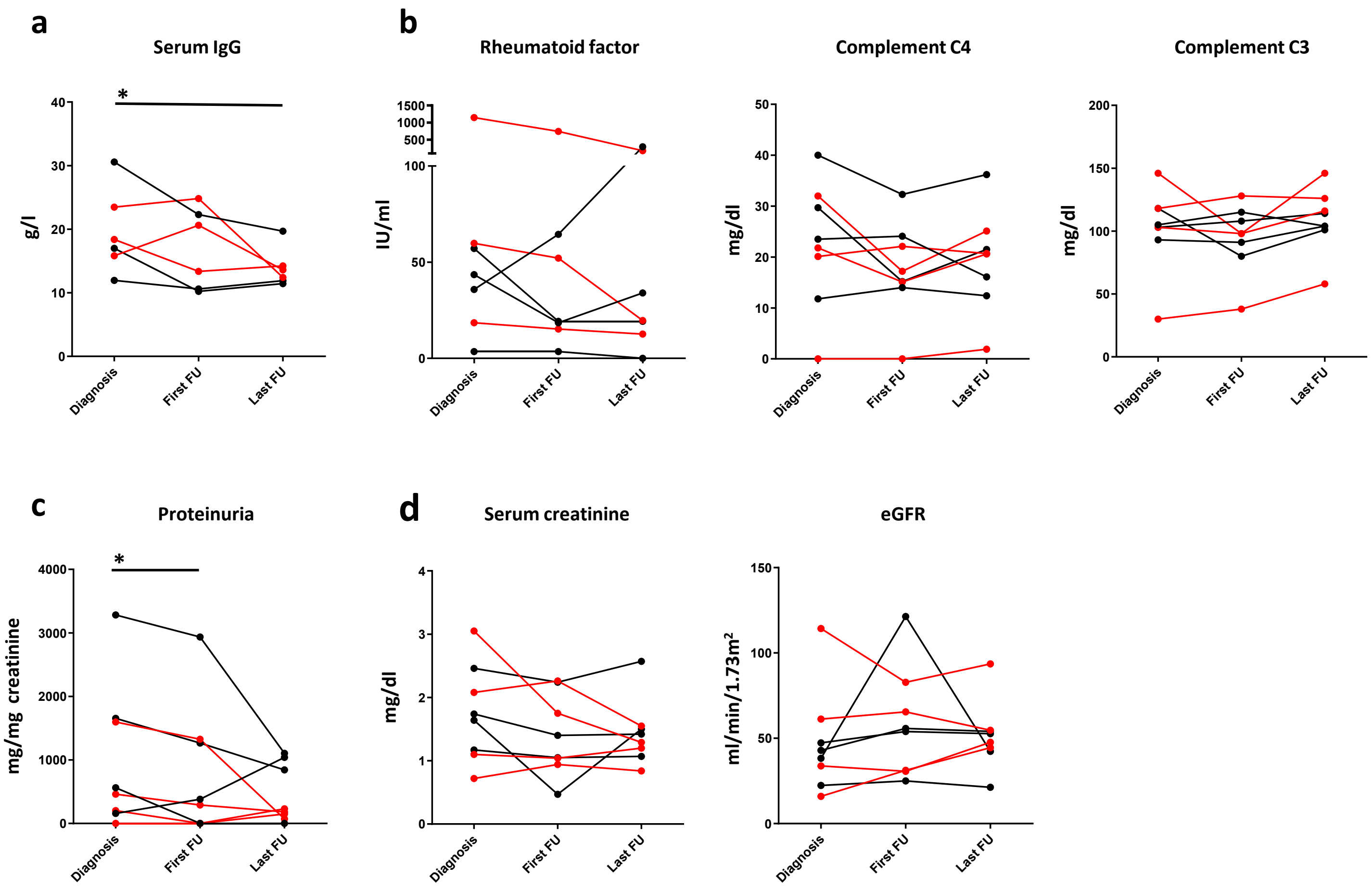

Supplementary figure S6. Response of clinical parameters to treatment

(a-d) Change of the indicated clinical parameters during treatment. The 4 patients with repeat biopsies are highlighted in red. All time points for all patients with SjD-TIN are plotted individually. Circles show individual patients. Estimated glomerular filtration rate (eGFR) is calculated using the 2009 CKD-EPI formula. *p<0.05.

Supplementary Figure S7. Response of non-T cell leukocytes to treatment

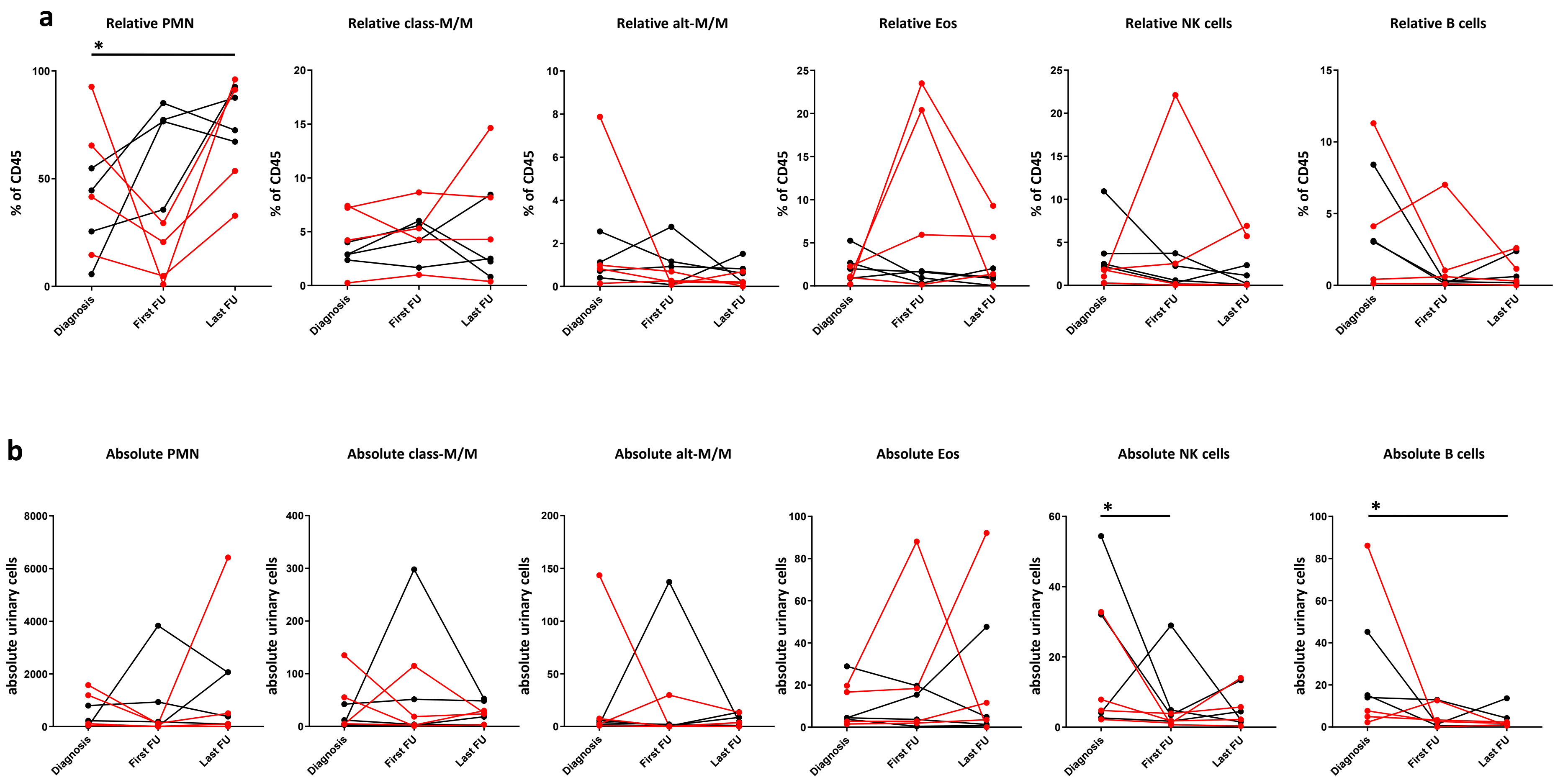

Supplementary figure S7. Response of non-T cell leukocytes to treatment

Change of the indicated (a) relative and (b) absolute urinary cell populations during treatment. The 4 patients with repeat biopsies are highlighted in red. All time points for all patients with SjD-TIN are plotted individually. Circles show individual patients. Absolute urinary cells are normalized to mg of urinary creatinine. *p<0.05.
